# Classifying behavior from short‐interval biologging data: An example with GPS tracking of birds

**DOI:** 10.1002/ece3.8395

**Published:** 2022-02-07

**Authors:** Silas Bergen, Manuela M. Huso, Adam E. Duerr, Melissa A. Braham, Todd E. Katzner, Sara Schmuecker, Tricia A. Miller

**Affiliations:** ^1^ Department of Mathematics and Statistics Winona State University Winona Minnesota USA; ^2^ U.S. Geological Survey Forest and Rangeland Ecosystem Science Center Corvallis Oregon USA; ^3^ Statistics Department Oregon State University Corvallis Oregon USA; ^4^ Bloom Research Inc. Los Angeles California USA; ^5^ West Virginia University Morgantown West Virginia USA; ^6^ Conservation Science Global, Inc. West Cape May New Jersey USA; ^7^ U.S. Geological Survey Forest and Rangeland Ecosystem Science Center Boise Idaho USA; ^8^ U.S. Fish and Wildlife Service Illinois‐Iowa Field Office Moline Illinois USA

**Keywords:** bald eagle, behavioral classification, biologging data, GPS telemetry, *K*‐means clustering, path segmentation, short‐interval data, unsupervised learning

## Abstract

1. Recent advances in digital data collection have spurred accumulation of immense quantities of data that have potential to lead to remarkable ecological insight, but that also present analytic challenges. In the case of biologging data from birds, common analytical approaches to classifying movement behaviors are largely inappropriate for these massive data sets.
2. We apply a framework for using *K*‐means clustering to classify bird behavior using points from short time interval GPS tracks. *K*‐means clustering is a well‐known and computationally efficient statistical tool that has been used in animal movement studies primarily for clustering segments of consecutive points. To illustrate the utility of our approach, we apply *K*‐means clustering to six focal variables derived from GPS data collected at 1–11 s intervals from free‐flying bald eagles (*Haliaeetus leucocephalus*) throughout the state of Iowa, USA. We illustrate how these data can be used to identify behaviors and life‐stage‐ and age‐related variation in behavior.
3. After filtering for data quality, the *K*‐means algorithm identified four clusters in >2 million GPS telemetry data points. These four clusters corresponded to three movement states: ascending, flapping, and gliding flight; and one non‐moving state: perching. Mapping these states illustrated how they corresponded tightly to expectations derived from natural history observations; for example, long periods of ascending flight were often followed by long gliding descents, birds alternated between flapping and gliding flight.
4. The *K*‐means clustering approach we applied is both an efficient and effective mechanism to classify and interpret short‐interval biologging data to understand movement behaviors. Furthermore, because it can apply to an abundance of very short, irregular, and high‐dimensional movement data, it provides insight into small‐scale variation in behavior that would not be possible with many other analytical approaches.

## Graphical abstract

We demonstrate the use of *K*‐means clustering to classify bird behavior using points from short time interval GPS tracks. We illustrate how these data can be used to identify behaviors and life stage‐ and age‐related variation in behavior. Our work has implications for understanding movement behaviors from GPS data.

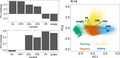

## 1 INTRODUCTION

Recent advances in digital data collection have spurred accumulation of immense quantities of data that have led to remarkable ecological insight (Hampton et al., 2013; Lewis et al., 2018; Thums et al., 2018). At the same time, these advances have created an “embarrassment of riches,” where analysis tools and technologies used in ecology have not kept pace with the quantity of data created (Katzner & Arlettaz, 2020; Kays et al., 2015; Williams et al., 2020). As a consequence, ecologists may under‐utilize their data due to a lack of awareness of, or lack of examples of, analytical tools available to manage and interpret the huge quantities of data being collected.

Animal tracking technologies, or “biologgers,” are an example of a digital data collection tool that has created unique opportunities for understanding animal ecology and behavior (Hussey et al., 2015; Kays et al., 2015). Biologgers collect data at time frames that allow researchers to identify and distinguish between different movement behaviors, which is a common objective in movement ecology. Some methods for identifying different behaviors only allow for a binary behavioral classification, for example, interpatch vs intrapatch search (Barraquand & Benhamou, 2008); intensive vs extensive search (Knell & Codling, 2012); foraging vs resting (Whitford & Klimley, 2019); and soaring vs flapping flight (Katzner & Arlettaz, 2020). Various other methods have been developed to more flexibly analyze animal movement data with the intention of understanding additional behavioral modes (Edelhoff et al., 2016; Gurarie et al., 2016). Common analytic approaches include Bayesian partitioning modeling (BPM; Calenge, 2006), behavioral change‐point analysis (BCPA; Gurarie et al., 2009), or variants of state‐space models (SSM) and hidden Markov models (HMM) (Gurarie et al., 2016; Langrock et al., 2012). These tools can be used to analyze covariates from biologging data alone, independent of external predictors (i.e., weather, topography, land cover), making them well suited to subsequent statistical analyses linking patterns in behavioral classes to patterns in the environment.

A defining feature of many, but not all of these tools is that, in ecology, they were initially applied to identify behavioral classes in telemetry data collected over relatively long (minutes to hours or days), and sometimes constant, time intervals. As technological advances now allow the collection and transmittal of telemetry data collected at ever shorter time intervals (<60 s), the nature of these high‐frequency data offers potential for new insights but also presents novel challenges (Kays et al., 2020). There are three specific features of short‐interval data in general that make them poorly suited to popular movement analysis methods like BPM, BCPA, SSMs, or HMMs. First, the sheer preponderance of data (on the order of millions of GPS fixes) calls for an analytic approach that is computationally efficient. Most movement analyses have been demonstrated only for much smaller sample sizes (e.g., fewer than 15,000 American bison fixes analyzed by a pooled HMM analysis in Langrock et al., 2012; 764 northern fur seal fixes analyzed with BCPA in Gurarie et al., 2009; 433 lamprey fixes analyzed with BPM, BCPA, and SMM/HMM in Gurarie et al., 2016). Second, short‐interval data often feature an abundance of short segments comprised of only a few positional fixes, resulting from data collection “bursts” that potentially occur sparsely over space and time. The strength of BCPA, HMM, and BPM is in their ability to characterize “change points” or “switches” in the behaviors of lengthy segments. However, it is futile to try to identify such change points in short‐interval segments that may comprise as few as three GPS fixes. Third, the time between short‐interval observations often is irregular, and HMMs and SMMs generally require that observations be made at regular time or space intervals (Patterson et al., 2017). Large data quantities are often dealt with by subsampling regularly spaced data, but given the ubiquity of very short segments in short‐interval data, this could result in a large loss of data. Recent methods have been developed using continuous‐time Markov chain approaches that can handle irregular space/time intervals between observations (Michelot et al., 2019; Wilson et al., 2018), but these methods center on understanding an animal's home range or utilization density and resource selection, not on defining behavioral modes. Recently, Adam et al. (2019) developed methods to extend HMM to multiple data streams occurring at different time scales, but even that approach assumes that each time scale was regularly sampled.

One additional challenge occurs when considering short‐interval biologging data from birds. Specifically, avian movement often occurs by flight. In this case, position is three‐dimensional, where location is defined not just on the *X*/*Y* plane but also the *Z* plane. This opens the possibility of a rich new suite of variables to define movement because velocity, acceleration, and deceleration have not just a horizontal component but also a vertical component. In fact, position on the *Z* plane, measured as altitude above ground, may be even more important for classifying behavior than is position on the *X*/*Y* plane (Sur et al., 2021). Most existing software implementations of popular movement analyses including BCPA (Gurarie, 2014), HMM (Michelot et al., 2016), or BPM (Calenge, 2006) assume the movement data comprise a two‐dimensional spatial component and a time component. Applications of HMM that have explored behavioral classification from vertical movement still only consider two dimensions (e.g., Phillips et al. (2015) analyze movement of tropical tuna characterized by depth and water temperature).

Characterizing behavior using short‐interval movement data from birds thus requires a computationally efficient method that can be applied to potentially millions of observations, an abundance of very short segment lengths, irregular time and space intervals between fixes, and high‐dimensional movement characteristics. Few, if any, tools have been developed that specifically address these issues. One appealing analytic approach for grouping individual trajectory points into behavioral clusters given these considerations is the *K*‐means clustering algorithm (Hastie et al., 2009). *K*‐means clustering is a popular unsupervised learning method that aims to identify clusters of data points with similar attributes. An appealing feature of *K*‐means clustering is that there is no limit on the dimensionality of the movement attributes, making it a very good option for clustering high‐dimensional movement data such as that from birds. In the animal movement literature, *K*‐means clustering has been applied to classify same‐state behavioral segments following BCPA (Zhang et al., 2015) but can also be applied directly to cluster individual points in a trajectory, allowing temporally consecutive locations to be assigned to different behaviors (Sakamoto et al., 2009; Van Moorter et al., 2010). This latter application of *K*‐means clustering is particularly attractive for short‐interval data because it does not require the presence of long segments that may be missing in short‐interval data sets. As it is relatively assumption‐free, *K*‐means clustering can be applied to correlated data without requiring estimation of an autocorrelation function. This is appealing as autocorrelation may be difficult to estimate with an abundance of very short temporally consecutive segments. Furthermore, *K*‐means algorithms are optimized for computational efficiency to speedily converge, even when applied to millions of data points, and are readily available in most standard statistical software.

Here, we use *K*‐means clustering to identify behavioral states associated with attributes measured at short intervals by biologgers. We apply this framework to GPS telemetry data collected at 1–11 s from bald eagles (*Haliaeetus leucocephalus*) in the state of Iowa, USA. We show how attributes associated with the *K*‐means clusters reflect biologically relevant behavioral states. Finally, to illustrate how such an analysis can provide insight into animal ecology, we analyze the sequentiality and relationship of these behaviors with intrinsic and extrinsic characteristics (e.g., Nathan, 2008), and we explore age‐ and stage‐related variation in the relationships. R code with an example data set to demonstrate the use of this approach is available in a GitHub repository.

## 2 MATERIALS AND METHODS

### 2.1 Data collection

From 2013 to 2019, we tagged 100 bald eagles in Missouri (*n* = 1), Oklahoma (*n* = 14), Illinois (*n* = 33), and Iowa (*n* = 52). These included 62 nestlings captured in the nest, 30 free‐flying birds captured using floating fish traps (Cain & Hodges, 1989) or net guns or cannon nets at deer carcasses (Bildstein & Bird, 2007), and 8 tagged at the time of release from rehabilitation facilities. All captured eagles were banded with a standard United States Geological Survey (USGS) federal bird band and outfitted with 70‐g Global Positioning System–Global System for Mobile communications (GPS‐GSM) telemetry devices (Cellular Tracking Technologies, LLC, Rio Grande, NJ, USA). The telemetry units were programmed to collect GPS data at ~3–10 s intervals, while the birds were in flight and at 15‐min intervals after the bird had stopped moving for 1 min (i.e., perching). That said, the actual interval between fixes varied around these programmed intervals. Whether or not the animal was moving was determined by manufacturer‐designed settings and based on parameters reported from an onboard accelerometer.

Telemetry devices were attached in a backpack style (Kenward, 1985) using a Teflon^®^ ribbon (Bally Ribbon Mills, Bally, PA, USA) harness. Weight of the device and harness was always <3% of body mass. We aged birds based on the length of the 8th primary (nestlings; Bortolotti, 1984) or based on plumage and molt characteristics (free‐flying birds; McCollough, 1989). We further classified free‐flying birds as juveniles (age ~5–12 months), subadults (age 1–4.5 years), or adults (>4.5 years). For additional details on animal capture, handling, permitting, and tagging, see Miller et al. (2019) and Schmuecker et al. (2020).

To understand within‐population variability in flight behavior, we manually assigned birds to one of three biological “stages” (fledged, local, and long distance). We used the term “fledged” to describe young eagles that had left the nest but that were still dependent on their parents. This period began when a nestling fledged and ended when it dispersed from the natal area. We determined that an eagle dispersed and was no longer dependent on its parents when it took a directed flight away from the natal area and did not return for >7 days (for additional details, see Miller et al., 2019). We used the term “long distance” to describe eagles that engaged in both migratory and directed dispersal movements (as defined in Miller et al., 2016; Poessel et al., 2016). Finally, we used the term “local” to describe birds that were neither fledged, migratory, nor dispersive (i.e., everything that did not fit into the other two categories). Many birds were tracked through multiple stages and across multiple years.

### 2.2 Pre‐analysis processing of data

Once collected, we filtered GPS data to remove outliers and points that diagnostic data suggested were of low precision (we refer to this as “first‐tier filtering”). Specifically, we filtered out data points for which the horizontal or vertical dilution of precision (HDOP or VDOP) was >10 or that had a 2‐dimensional GPS fix (as opposed to 3‐dimensional). HDOP and VDOP are confidence measures of the GPS horizontal and vertical positions, respectively, where lower values are equal to higher confidence. We also calculated the altitude above ground (AGL) of every location by subtracting the value of a 30‐m digital elevation model (Gesch et al., 2002) at that location from the GPS‐determined altitude above sea level (ASL), and we filtered out all data points in which AGL < −50. Finally, we carefully examined outliers with respect to altitude, speed, and other metrics (e.g., time between points, distance between points), and we removed obviously anomalous points. For additional details on our approach to data management and filtering, see Poessel et al. (2018).

We used the GPS data to identify or calculate values for a set of six focal variables (Table 1) known to effectively describe eagle flight (for details on these variables, see Katzner et al., 2015). Two of these, instantaneous speed (KPH) and meters above ground level (AGL), were characteristics of a single GPS data point. The other four were derived from up to three sequential points (calculations in Table 1). These include speed between two points (distance/time, Sn), the rate of gain in altitude between two points, that is, vertical rate (m gained/s, Vr), the absolute value of vertical rate (|Vr|), and the absolute value of the turn angle (computed over three sequential points, |Angle|, in radians, package moveHMM, Michelot et al., 2016). Thus, “movement” measured at each GPS point was defined with a 6‐dimensional suite of focal variables.

**TABLE 1:** Definitions of biologging data collected from bald eagles and subsequent variables derived from these data

Variable	Definition	Calculation	Used in clustering?	Square‐root transformed?
*x*	UTMEasting	From biologger	No	–
*y*	UTMNorthing	From biologger	No	–
*z*	Altitude above sea‐level (m)	From biologger	No	–
Date	Date	From biologger	No	–
Time	Time	From biologger	No	–
KPH	Instantaneous speed (k/h)	From biologger	Yes	Yes
Sn	Horizontal distance between consecutive points at *T_i_* and *T_i_*_−1_ divided by change in time (*T_i_* − *T_i_*_−1_) (m/s)	[(*x_i_* − *x_i_*_−1_)^2^ + (*y_i_* − *y_i_*_−1_)^2^]^1/2^/(*T_i_* − *T_i_*_−1_)	Yes	Yes
AGL	Above ground level (m)	AGL = (*z_i_* − DEM*_i_*)	Yes	Yes
|Angle|	Abs. value of turn angle (radians)		Yes	Yes
Vertical rate	Mean vertical velocity, change in altitude/change in time between consecutive sample points (m/s)	(*z_i_* − *z_i_*_−1_)/(*T_i_* − *T_i_*_−1_)	Yes	No
|Vertical rate|	Absolute value of vertical rate (m/s)		Yes	Yes

Note

Whether each variable was used in *K*‐means classification and, if so, whether it was first transformed is also indicated.

After focal variables were defined, we conducted a series of additional filtering steps (“second‐tier filtering”). First, we removed points that had any missing values for any of the six focal variables. Second, we removed points that were >11 s apart. We chose the 11‐s threshold based on inspection of our data set, in which ~97% of the first‐tier filtered data were ≤11 s apart. This requirement also ensured that the derived focal variables (Sn, |Angle|, Vr, and |Vr|) were measured over similar time spans. Third, we filtered out points that were not part of a segment of at least three consecutive points, each ≤11 s apart.

The *K*‐means algorithm tends to be more effective at identifying meaningfully distinct clusters if distributions of variables are not highly skewed. Likewise, it is important that the variables used in the clustering are on a similar scale, such that the Euclidean distance metric underlying the algorithm is not driven by variables with larger standard deviations. Accordingly, we visualized the distributions of the focal variables with histograms and applied a square‐root transformation to variables that had distributions with visually apparent skew. Subsequently, we centered and scaled all variables by subtracting the mean and dividing by standard deviation.

### 2.3 *K*‐means clustering

The *K*‐means algorithm is a simple approach for partitioning a data set into *K* distinct, nonoverlapping clusters (James et al., 2013). We briefly describe the algorithm here in the context of behavioral classification by assigning each of *N* GPS points to one of *K* distinct behavioral modes.

Let $x_{i}=\left\{x_{i1},x_{i2},\ldots ,x_{\mathrm{ip}}\right\}$ denote the p quantitative data values of covariates for the GPS point at time $T_{i}$; i=1,…,N, for N GPS points. The algorithm is initialized by assigning a random cluster membership of 1 through *K* to each of the *N* GPS points. Let $C_{k}$ denote the set of points in cluster *k*, k=1,…,K, and $\left|C_{k}\right|$ the number of points in cluster $C_{k}$. Then, ${\bar{x}}_{\cdot jk}=\frac{1}{\left|C_{k}\right|}\sum_{x_{i}\in C_{k}}x_{\mathrm{ij}}$ is the mean of the jth covariate among all points in $C_{k}$, and $m_{k}=\left\{{\bar{x}}_{\cdot 1k},{\bar{x}}_{\cdot 2k},\ldots ,{\bar{x}}_{\cdot pk}\right\}$ is the centroid of $C_{k}$. The algorithm proceeds as follows:

1. For each point, $x_{i}$, calculate $d_{i1}\ldots d_{\mathrm{iK}}$ as the squared Euclidean distance of GPS point $x_{i}$ from centroid $m_{k}$:

$$d_{\mathrm{ik}}=\sum_{j=1}^{p}{\left(x_{\mathrm{ij}}‐{\bar{x}}_{\cdot jk}\right)}^{2}.$$

2. Reassign each point, $x_{i}$, to the cluster with the closest centroid; equivalently, to the $C_{k}$ for which $d_{\mathrm{ik}}$ is the smallest:

$$C_{k}=\left\{x_{i}:\min\left\{d_{i1},d_{i2},\ldots ,d_{\mathrm{iK}}\right\}=d_{\mathrm{ik}}\right\}.$$

3. Compute the new centroids $m_{k}$ of the newly assigned clusters for k=1,…,K.

The objective of the algorithm is to minimize the total within‐cluster sum of squared Euclidean distances, $\text{WSS}=\sum_{k=1}^{K}\sum_{i=1}^{N}d_{\mathrm{ik}}$, for all possible definitions of clusters. The algorithm iterates between steps 1–3 until the centroids and assigned cluster memberships do not change (i.e., the within‐cluster sum of squares is minimized). It is good practice to specify numerous “starting points” (initial random assignments of the cluster memberships and hence initial centroids) to ensure that the resulting cluster definitions are not a function of the initial centroids and that a global minimum for WSS has truly been found for the given value of *K*. We used the kmeans() function in R to implement the clustering (R Core Team, 2020). See Data Availability for access to example R code.

### 2.4 Choosing *K*

In order to apply the *K*‐means algorithm, one must first specify *K*, the number of clusters to identify. The algorithm will then classify each data point as belonging to one of the *K* clusters. Subsequently, one must determine the value of *K* that best describes uniquely distinct groupings. A common, simple method for choosing *K*, often referred to as the “elbow method,” involves looking at a plot of the total within‐cluster sum‐of‐squares (WSS) of clustered data as a function of *K* (Hastie et al., 2009). The WSS measures the total squared distance of all points from the centroid of their assigned clusters. As *K* increases, the WSS will decrease, and there is often a value of *K* that corresponds with a “kink” or “elbow” in the plot before which the WSS steeply descends and after which the WSS levels off. This “elbow” indicates a *K* for which a larger number of clusters do not reveal additional meaningfully distinct groupings.

It is possible for the elbow method to be ambiguous about the optimal *K*. An alternative method for choosing *K* is the average silhouette method (Rousseeuw, 1987). The “silhouette width” of a data point measures how close it is on average to members of its own cluster relative to members of the closest neighboring cluster. A silhouette width of 1 indicates a very confidently clustered point; widths of 0 indicate a point on the border of two clusters; and negative widths indicate a point that may be in the incorrect cluster. Averaging the silhouette widths across the entire data set provides a metric that can be compared across different values of *K*, with higher average silhouette values indicating better choice of *K*. However, calculating the silhouettes for all data points simultaneously requires computing the *N* × *N* distance matrix, which is not computationally feasible for a data set of over one million observations. A work‐around is to bootstrap the average silhouette by sampling *N_boot_* ≪ *N* points with replacement from the original data set. The average silhouette can then be computed on the bootstrap sample for each value of *K*. Repeating this process *B* times yields *B* bootstrapped average silhouettes for each *K*, which can be plotted as a function of *K*. The values of *K* that tend to yield the highest average silhouettes across the *B* bootstrap samples are candidates for the optimal number of clusters.

There may be cases in which the bootstrapped average silhouettes suggest two or more optimal values of *K*. In these situations, it can be useful to reduce dimensionality of the covariate space by way of a principal component analysis and create a biplot of the first two principal components (PCs), color‐coded by *K*. Input data into the principal component analysis are not the bootstrapped data, but instead are the same as those used in the *K*‐means clustering as described above (i.e., the GPS data in Table 1, transformed and standardized). The first two PCs explain the largest proportion of variability in the covariates. Creating a “lineup” of these biplots for each value of *K* may provide helpful insight into the value of *K* that best describes distinct, biologically relevant behaviors.

### 2.5 Data analysis and behavioral interpretation

For our example analysis on telemetry data for bald eagles, we carried out *K*‐means clustering for $K\in \left\{2,\ldots ,7\right\}$ with 10 randomly chosen initial cluster assignments for each *K*. We used both the elbow method and the bootstrapped silhouette method along with a biplot lineup to identify an optimal value for *K*. For the silhouettes, we took *B* = 1000 bootstrap samples each of size 10,000 and averaged the silhouette widths across these 10,000 points for each value of *K*. The clusters assigned to points in each bootstrap sample were defined using the entire data set; clusters were not redefined for each bootstrap sample. Given the size of our data set and to avoid overplotting, we investigated biplot lineups of several individual birds separately rather than plotting biplots of all birds together and verified the similarity of the biplots across individual birds. Subsequently, we examined the behavioral characteristics of each cluster identified by the *K*‐means approach. To do this, first, we evaluated the relationship between these clusters and each focal covariate using a series of boxplots and, using these relationships, we determined a behavioral mode for each cluster. Second, we evaluated patterns in sequentiality of GPS data. To do this, we identified *behavioral subsegments*. These were defined to be consecutive points ≤11 s apart in which all points within the subsegment had been classified as belonging to the same behavioral cluster. We determined the durations of these behavioral subsegments and investigated the sequentiality in duration and classification of behavioral subsegments to gain further insight into the clusters and patterns of behavior. Third, to illustrate how this approach provides insight into within‐population variability in flight behavior, we evaluated age‐ and stage‐related variation in the frequency of behavioral occurrence of eagles by investigating plots of the relationship between eagle age and biological stage and behavioral classification.

## 3 RESULTS

### 3.1 Tracking data

We collected ~4.2 million GPS data points from bald eagles within Iowa. Of the 100 eagles captured in the Midwest and Great Plains, 57 provided data within Iowa and at time intervals useful for this study (Table 2). These included 28 nestlings, 39 juveniles, 23 subadults, and 11 adults (many birds were monitored across more than one year and thus may be represented in counts of multiple age classes). About three‐quarters of these data were collected from birds in the local stage (Table 3A).

**TABLE 2:** Number of bald eagles tagged and number of individuals tracked with GPS telemetry, organized by bird age

Bird age	Number captured	Individuals tracked
Nestling	42	28
Juvenile	3	39
Subadult	2	23
Adult	10	11
Total	57	101

Note

Many birds were tagged as nestlings but since nestlings do not fly, none of their tracking data were relevant to the analysis presented here. Most birds were captured in one stage and tracked into one or two other life stages; hence, the counts for each age class older than nestling are larger than the numbers of individuals tagged in each age class.

**TABLE 3:** Summary statistics describing (A) GPS telemetry points collected from bald eagles and classified into behavioral modes, by life stage and year of the study and (B) segments of classified GPS data subsequently used to illustrate effectiveness of *K*‐means clustering to understand animal movement and characteristics of flight behavior

(A)				
Year	Life stage			Total
	Fledgling	Locally moving	Dispersal/migration	
2016	0	2795	2901	5696 (0.3%)
2017	60,767	142,953	37,077	240,797 (11.5%)
2018	86,615	184,105	47,906	318,626 (15.2%)
2019	151,934	419,847	123,045	694,826 (33.2%)
2020	0	788,773	44,304	833,077 (39.8%)
Total	299,316 (14%)	1,538,473 (74%)	255,233 (12%)	2,093,022

(B)		
	Segment^a^ length measured in	
	GPS points	Seconds
Minimum	3	2
25th percentile	4	19
Median	5	27
75th percentile	11	69
Maximum	3538	21,222

^a^ Each ≥3 consecutive points of ≤11 s apart.

### 3.2 Pre‐analysis processing

After first‐tier filtering, we retained 2,793,220 high‐precision GPS data points. After second‐tier filtering, we retained 2,093,022, or 36,720 ± 44,190 points per bird ($\bar{x}$ ± *SD*; range: 27–187,570). Creating segments of consecutive points ≤11 s from each other resulted in 77,259 segments that ranged in length from 3 points and 2 s in duration (the minimum segment length) to >3500 points and 354 min (Table 3B). Among the 57 birds we analyzed, the number of segments per bird ranged from 2 to 8220 with a median number of segments per bird of 1016. The vast majority of these segments were short: 90% of segments were ≤38 points and ≤240 s; 75% were ≤11 points; and half of the segments were ≤5 points.

**FIGURE 1:**
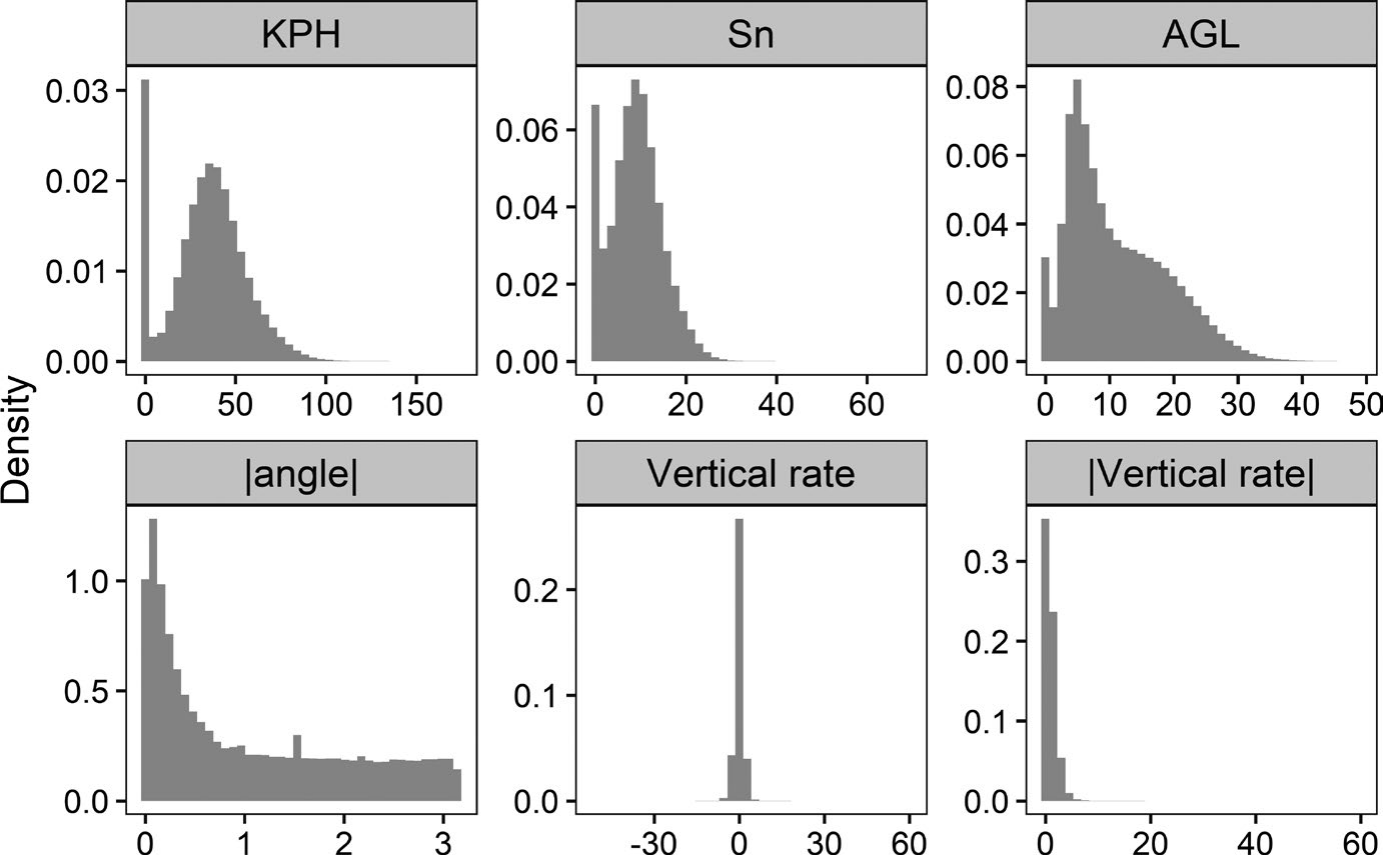
Distributions of eight focal variables associated with GPS data collected from bald eagles in Iowa, USA. Focal variables were standardized (after square‐root transform when necessary to reduce skewness) then used as input into a *K*‐means cluster analysis to classify flight behavior of these birds. See text for details on analysis

Distributions of the six focal variables associated with GPS points are shown in Figure 1. Speed measurements at GPS points were bimodally distributed, with one peak at zero (for motionless birds) and a second at ~45 kph (KPH) and ~10 kph (Sn). In contrast, distributions of AGL, |Angle|, and |Vr| all were all positively skewed with peaks at or near zero, while Vr was symmetrically centered at zero. We applied a square‐root transformation to KPH, Sn, AGL, |Angle|, and |Vr|. We did not transform Vr. All variables (transformed or not) were subsequently standardized to mean = 0 and variance = 1 for clustering.

### 3.3 *K*‐means clustering

**FIGURE 2:**
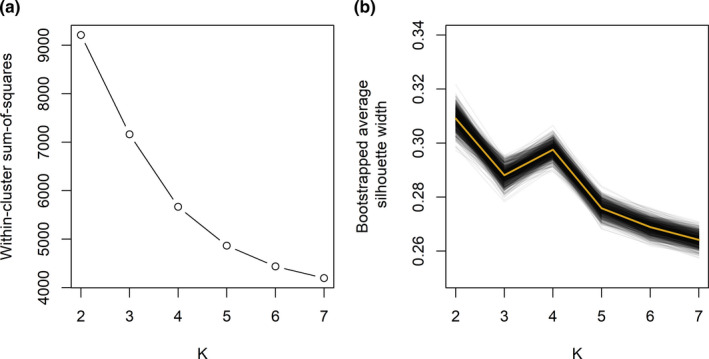
Plots of (a) within‐cluster sum‐of‐squared distances between each point and the cluster centroid; and (b) bootstrapped average silhouette width as a function of number of specified clusters *K*. In (b), the gold line represents the mean across all 1000 bootstrap samples of the average silhouette widths. Cluster centroids were determined by a *K*‐means analysis of standardized GPS telemetry data collected from bald eagles in Iowa, USA. See main text for additional details on data collection and analysis

**FIGURE 3:**
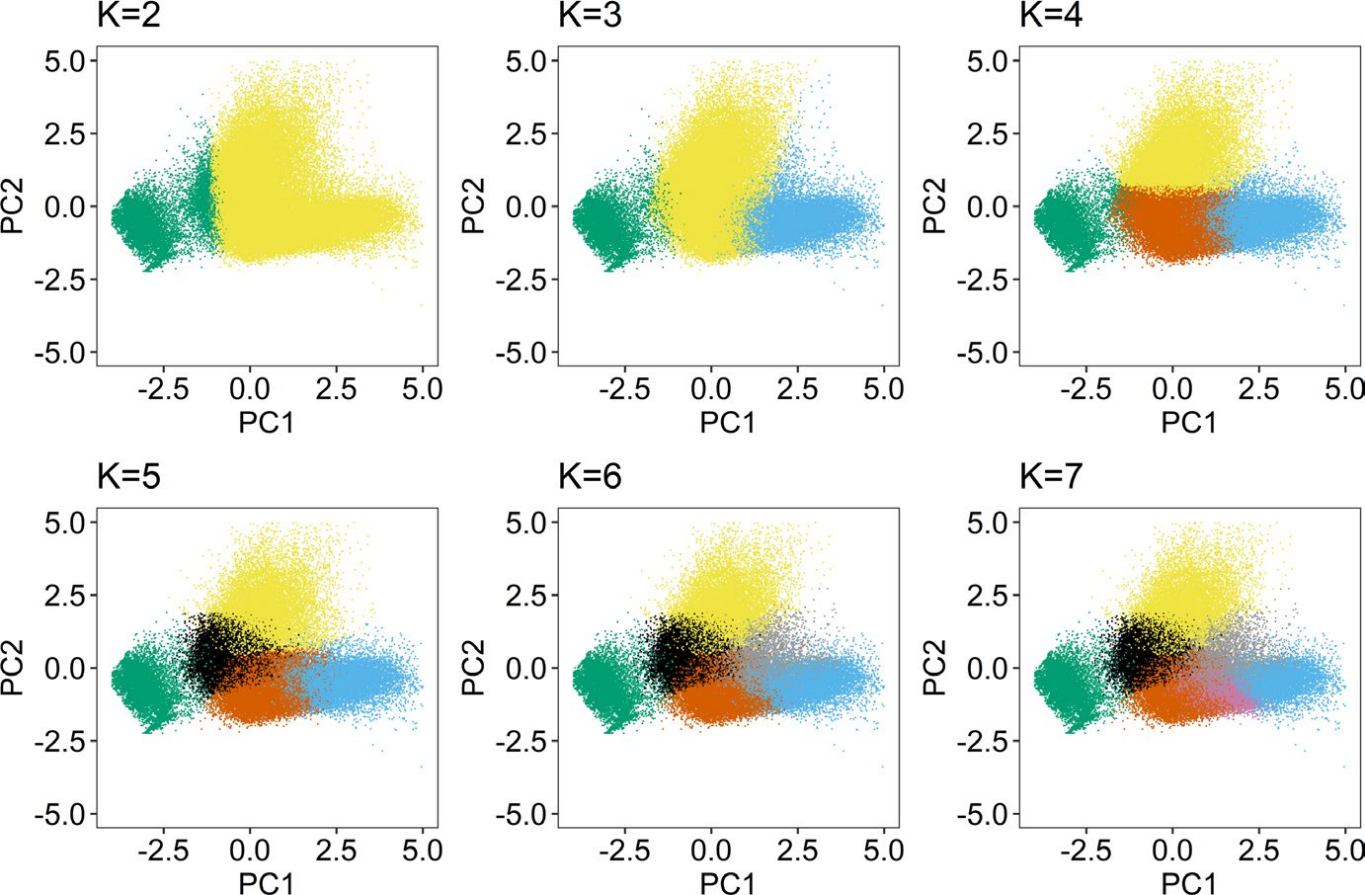
Examples of biplots of the first two principal components of raw GPS telemetry data collected from a single bald eagle color‐coded by cluster membership for *K* ∈ {2, 3, 4, 5, 6, 7}. Input variables are those used in the *K*‐means clustering as described in the main manuscript (i.e., the GPS data in Table 1, transformed and standardized). Making these plots for each value of *K* in addition to the bootstrapped average silhouette widths provides additional insight into which number of *K* is most appropriate. In each biplot, the number of colors present equals the value of *K* indicated

The elbow method did not suggest that any single number of clusters was optimal (i.e., there was not a clearly defined “elbow”; Figure 2a). In contrast, plotting the bootstrapped average silhouettes widths across *K* suggested that *K* = 2 and *K* = 4 yielded the highest average silhouettes (Figure 2b). Whereas *K* = 2 tended to have slightly higher average silhouettes than *K* = 4, biplots of the first two principal components (PCs) indicated that *K* = 2 resulted in one small and one much larger cluster (Figure 3). Subsequent investigation indicated the small cluster was perching points (see *Behavioral interpretation of clusters*) while all in‐flight points were grouped into the large cluster. According to Figure 2b, *K* = 4 was clearly the optimal choice for breaking this larger flight cluster into three smaller in‐flight modes. As we were interested in clustering different flight modes rather than just flight vs nonflight, we selected *K* = 4 as the optimal number of clusters. The biplot structures in Figure 3 held when evaluating biplots from other individual birds and from multiple birds simultaneously.

**FIGURE 4:**
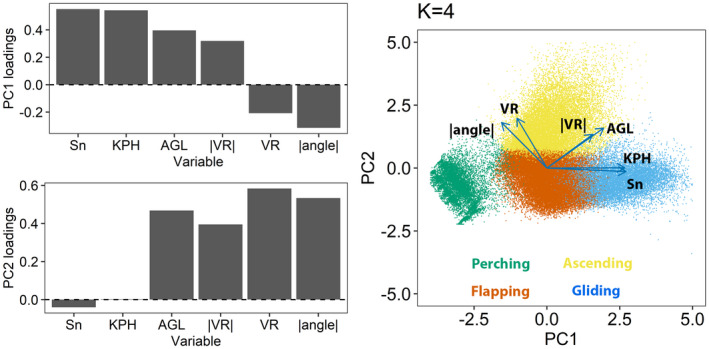
Loadings and biplot from principal component analysis of GPS telemetry data collected from bald eagles in Iowa, USA. Input data were the 6 focal variables shown in Table 1 in the main text. The first two components accounted for, respectively, 45% and 21% of the total variability in the covariates. Points on biplot are from a representative bird and are color‐coded by cluster membership assignment for *K *= 4 clusters

The most important focal variables for differentiating clusters in the first PC dimension were the two velocity variables (KPH and Sn; Figure 4). The other four variables (Vr, |Vr|, AGL, and |Angle|) were all more important for the second PC dimension. The first two PCs cumulatively accounted for 66% of the total variability in the covariates.

### 3.4 Behavioral interpretation of clusters

**FIGURE 5:**
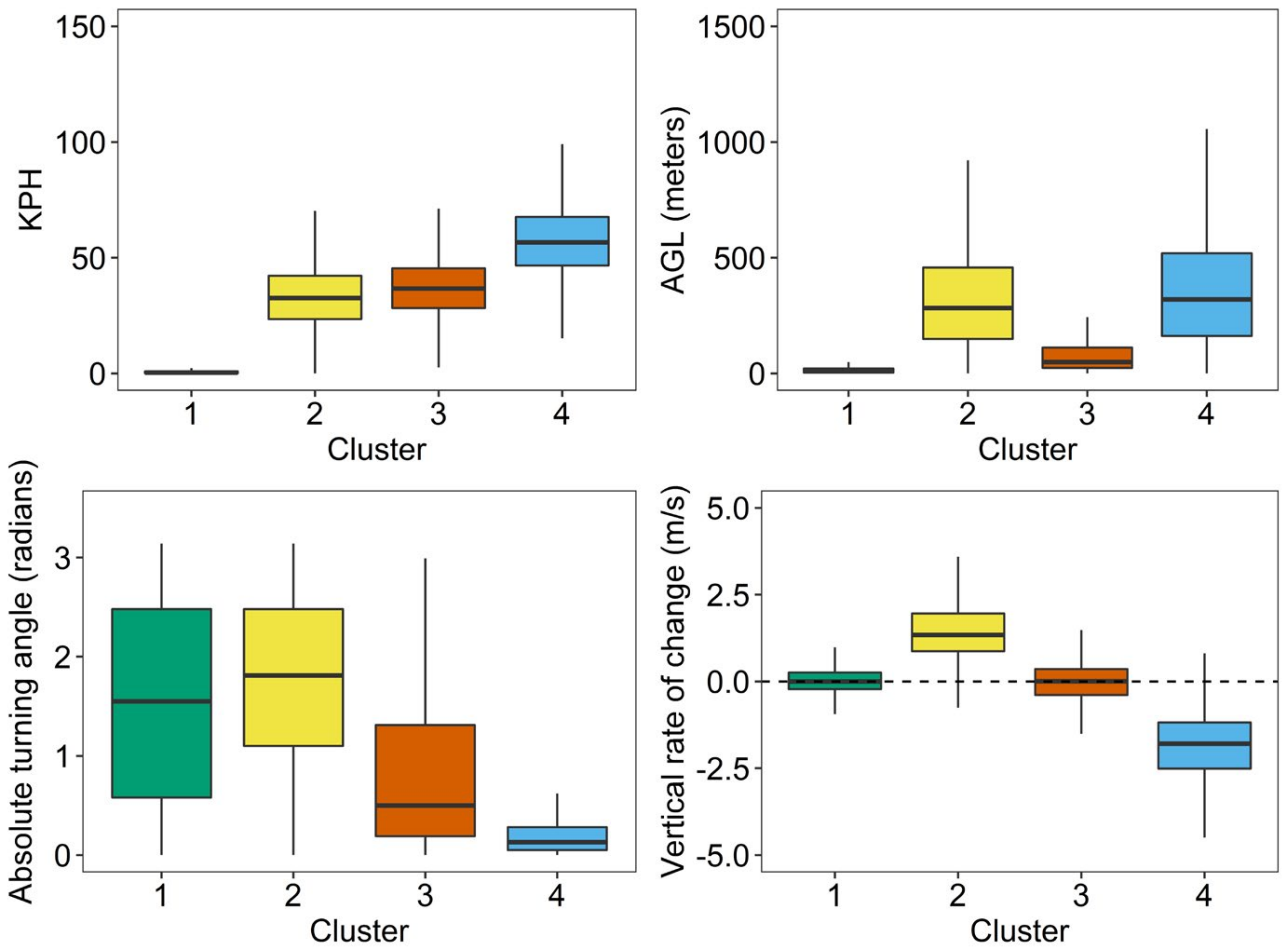
Boxplots of variables relevant to clustering short‐interval GPS data from bald eagles. Boxes visualize 25th, 50th, and 75th percentiles. Whiskers extend either to the most extreme data value or 1.5 * IQR (IQR = 75th–25th percentile) from the nearest quartile, whichever is closest. Variables chosen were those with high factor loadings from a principal components analysis of GPS variables used in *K*‐means clustering with *K* = 4, as described in the main text. AGL, altitude above ground level; KPH, kilometers per hour

Once clusters were defined, we then identified behavioral modes for each. The distribution of the focal variables among the clusters suggested movement characteristics that appeared to be associated with specific behavioral modes (Figure 5). For example, points in cluster 1 tended to have speeds near zero, were at low altitude above ground, had little vertical change, and had highly variable turning angles (Table 4; Figure 5). This pattern is consistent with a bird being motionless on the ground or in a tree (i.e., “perching”). In such a setting, the highly variable turning angles were generated by repeated small variations in GPS locations (i.e., GPS error). Points in cluster 2 had moderate velocities, positive vertical rates, and tortuous flight paths (high values of absolute angle) and occurred at high altitudes. We therefore interpreted points in cluster 2 to be indicative of a bird gaining altitude in an updraft (“ascending”). Points in cluster 3 were of moderate velocity and variable tortuosity while tending to be level and close to the ground. We deemed these points to be characteristic of “flapping” flight. Finally, points in cluster 4 exhibited fast velocities and straight (nontortuous) flight paths indicated by absolute angle values near zero. These points also occurred at high altitudes and were in descending flight (negative vertical rates). We therefore interpreted points in cluster 4 as indicative of a bird gliding from a thermal (“gliding”).

**TABLE 4:** Qualitative definition of clusters defined by *K*‐means clustering of GPS telemetry data collected from bald eagles

Cluster	Velocity	AGL	Angle	Vertical rate	Flight mode
1	Very slow	Very low	Highly variable	Level	Perching
2	Moderate	High	Angling	Ascending	Ascending
3	Moderate	Low	Variable	Level	Flapping
4	Fast	High	Straight	Descending	Gliding

Note

**FIGURE 6:**
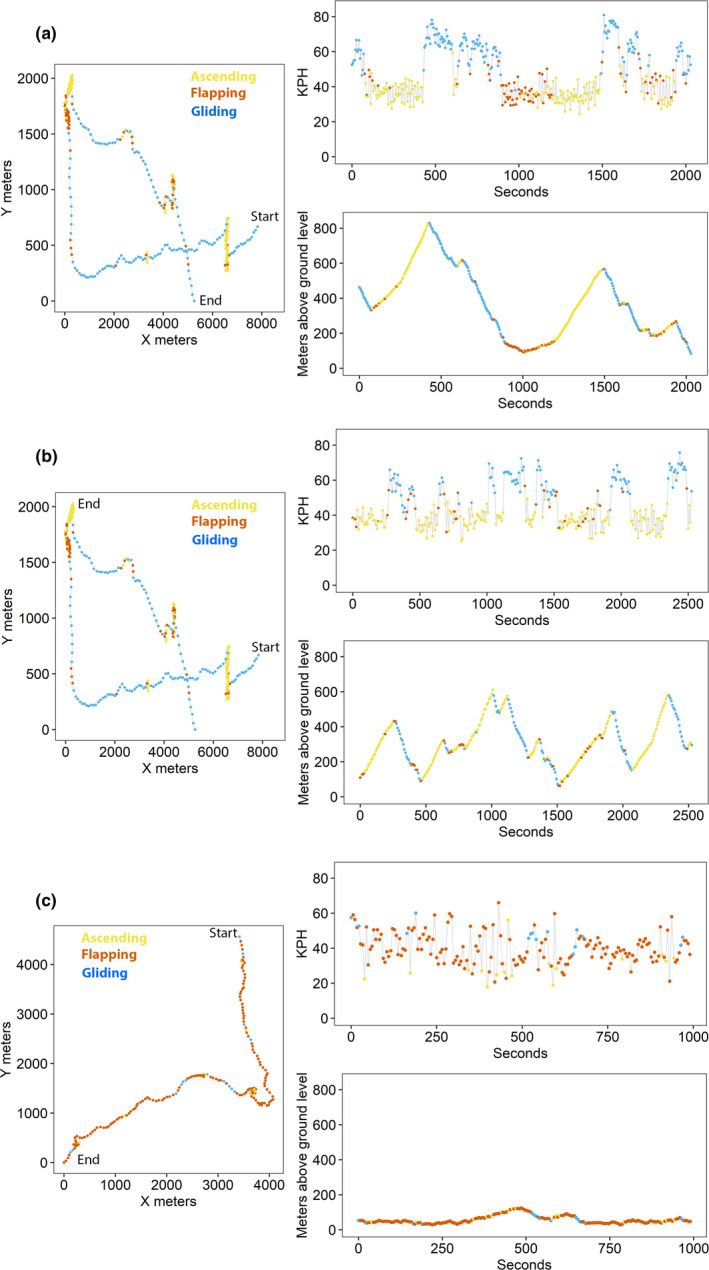
Plots showing details of movements and behaviors of bald eagles. Movement data were collected by GPS telemetry collected at short time intervals. GPS data were then assigned to behavioral categories via *K*‐means clustering. In each panel of the figure, the left‐most plot shows the map of the bird's movement, plotted on a UTM scale. The top right plot shows the flight speed of each point plotted sequentially over time. The bottom right plot shows the altitude above ground level, also plotted sequentially over time. In all three panels, points are color‐coded based on the behavioral mode identified by the *K*‐means clustering

Velocity was measured both by instantaneous speed measured by the GPS and by distance divided by time between sequential GPS data points. Altitude above ground level (AGL) is measured by the difference between altitude above sea level, measured by the GPS, and ground elevation measured by a digital elevation model (see main text for details). Angle and vertical rate were calculated over three GPS data points. Flight mode label was assigned by experts with a strong background in eagle ecology and behavior. Empirical trends for each of these measurements, by cluster, are shown in Figure 6.

Plots of sample flight paths illustrated these behavioral classifications (Figure 6). Ascending flight covered little ground and data points were clustered together (Figure 6a), resulting in slow (Figure 6a–c), climbing (Figure 6a,b) flight. Longer segments of consecutive ascending points tended to be followed by gliding flight (Figure 6a,b). The gliding flight that followed ascending flight resulted in sustained directional flight in which GPS data were spatially far apart and that were accompanied by rapid loss of altitude (Figure 6a,b) at high speeds (Figure 6a–c). Both ascending and gliding flight were occasionally interrupted by flapping behaviors. These interruptions tended to be at lower or occasionally intermediate altitudes, and they regularly occurred after gliding but before ascending flight. Like gliding flight, flapping flight tended to be straighter but at slower velocity (Figure 6a,b) and lower altitude (Figure 6c).

### 3.5 Relationship of behavior with age and movement stage

**FIGURE 7:**
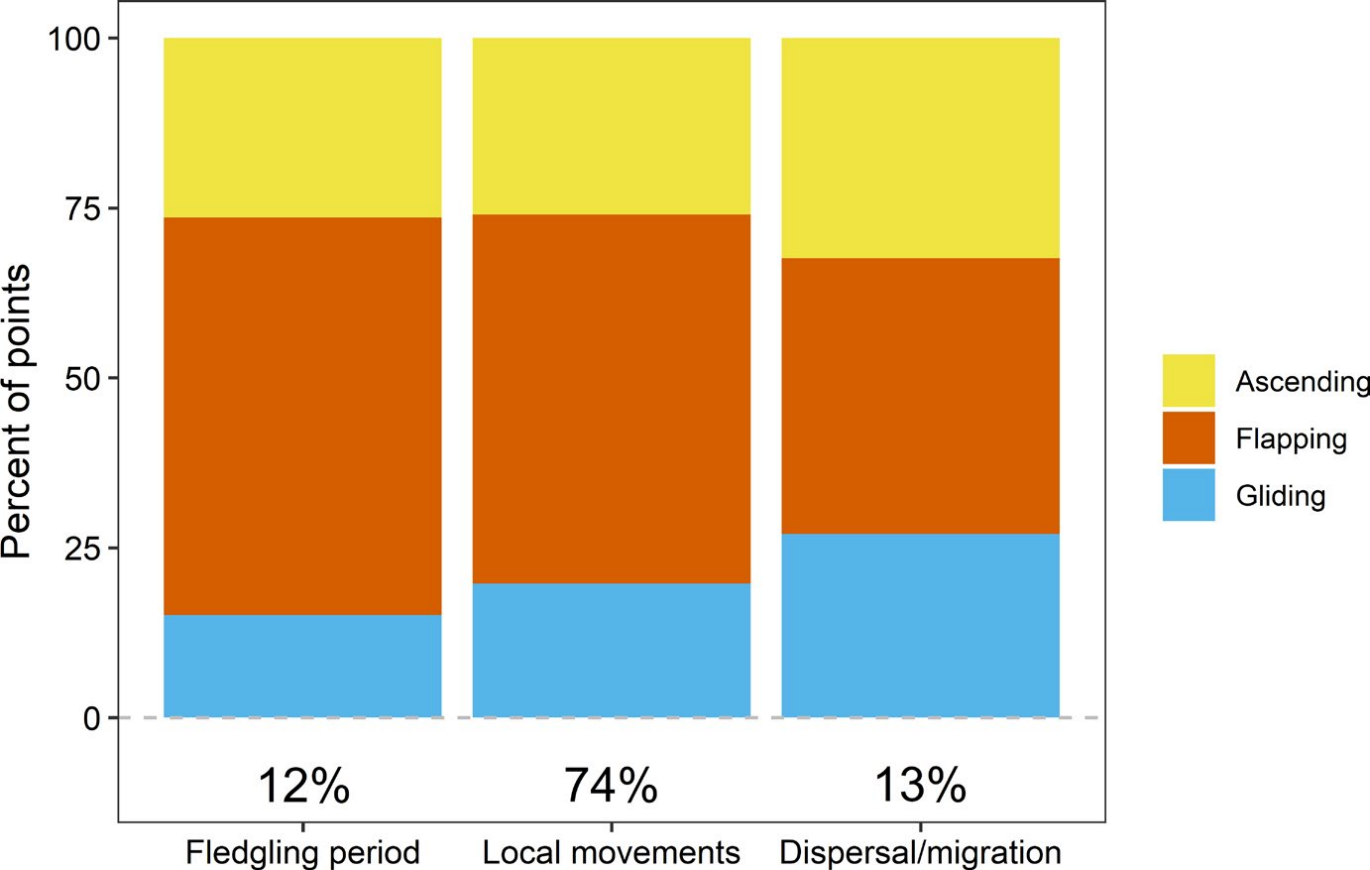
Relationship of behavioral classifications with life stages of eagles. Behavioral classifications were assigned by experts with a strong background in eagle ecology and behavior and associated with *K*‐means clusters of GPS telemetry data from bald eagles in Iowa, USA. Life stages of eagles were determined by gross movement characteristics of birds (i.e., were their movements migratory or local in nature). See main text for additional details on clustering and assignment to life stages. Bold numbers under bars indicate marginal percent of points in each flight stage

**FIGURE 8:**
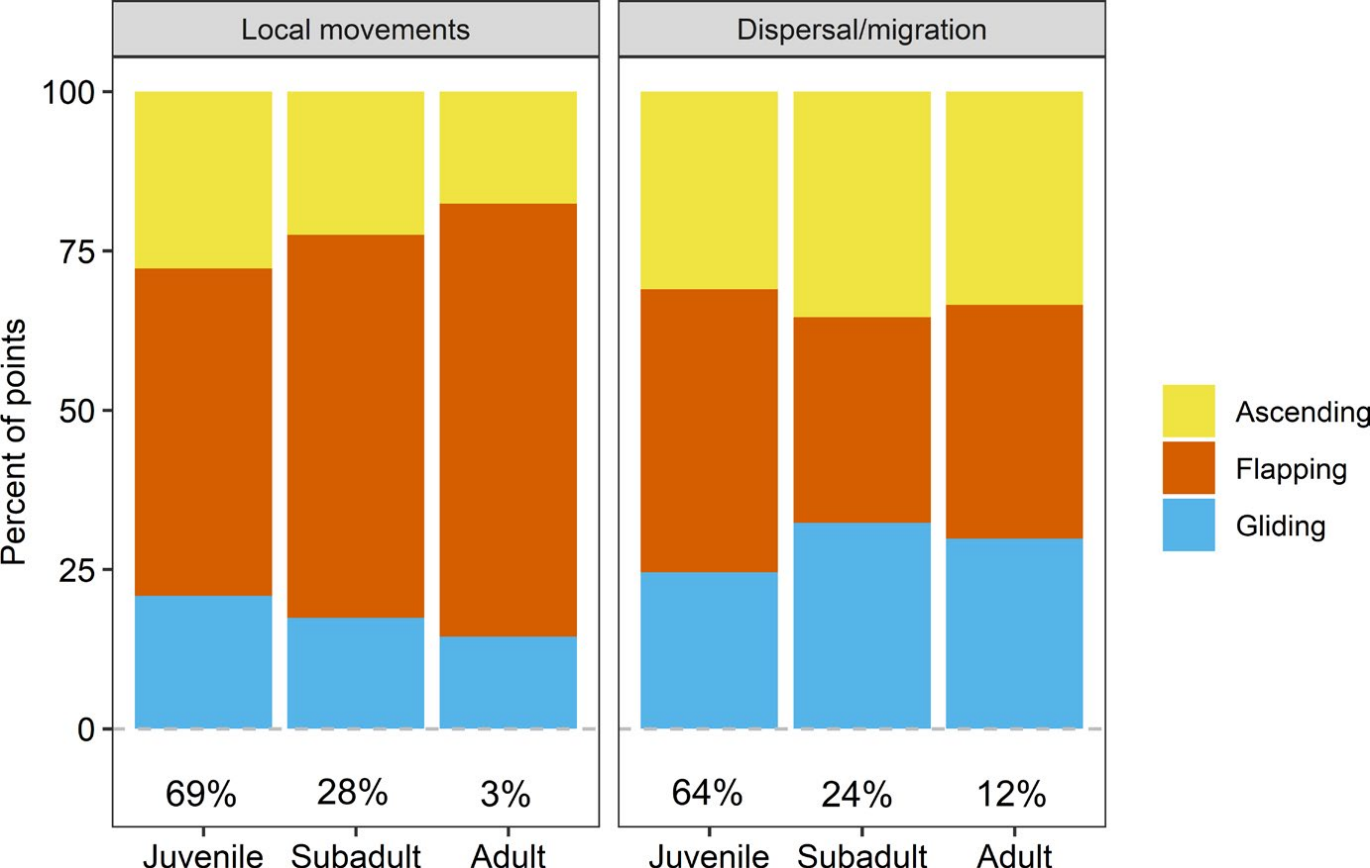
Relationship of behavioral classifications with age classes and life stages of eagles. Behavioral classifications were assigned by experts with a strong background in eagle ecology and behavior and associated with *K*‐means clusters of GPS telemetry data from bald eagles in Iowa, USA. Life stages of eagles were determined by gross movement characteristics of birds (i.e., were their movements migratory or local in nature). Ages were estimated when eagles were marked. See main text for additional details on clustering and assignment to age classes and life stages. Bold numbers under bars indicate marginal percent of points in each age class and of each flight stage

We detected, among age and stage classes of eagles, substantial differences in frequency of occurrence of each behavior type (Figures 7 and 8). Recently fledged birds, which have weaker flying skills than older birds, were less frequently in ascending or gliding flight and more often in flapping flight than were other birds (Figure 7). Eagles engaged in dispersal or migration more frequently exhibited high‐altitude behaviors (ascending and gliding) than did local birds. Among birds engaged in local movements, older eagles were more likely to exhibit flapping flight and less likely to exhibit ascending or gliding flight than were younger ones (Figure 8). Age differences were much less pronounced for migrating or dispersing birds than for birds moving only locally.

### 3.6 Analysis of behavioral subsegments

After clustering, we identified 587,425 behavioral subsegments, each consisting of consecutive points ≤11 s apart in which all points within the subsegment had been classified as belonging to the same behavioral cluster. Of these, 499,124 were nonperching subsegments (Table 5). About half of nonperching behavioral subsegments consisted of a single point (0 s subsegments). Ascending flight had the highest prevalence of subsegments consisting of a single GPS location (62% of ascending subsegments) and flapping had the highest prevalence of longer subsegments (22% of gliding subsegments were >22 s).

**TABLE 5:** Frequency of short‐, medium‐, and long‐duration behavioral subsegments of consecutive points belonging to one of three in‐flight behavioral modes of bald eagles

	Duration proportions of behavioral subsegments			Count
	0 s	0–22 s	>22 s	
Ascending	0.62	0.24	0.14	154,087
Flapping	0.42	0.36	0.22	242,957
Gliding	0.51	0.22	0.19	102,080
Count	249,582	154,436	95,106	499,124

Note

Behavioral modes were assigned by experts with a strong background in eagle ecology and behavior and were associated with *K*‐means clusters of GPS telemetry data. Subsegments of 0 s indicate a single point. Subsegments of up to 22 s are most frequently indicative of 3 GPS points (maximum inter‐fix interval was 11 s). Counts are numbers of behavior subsegments of each category.

**FIGURE 9:**
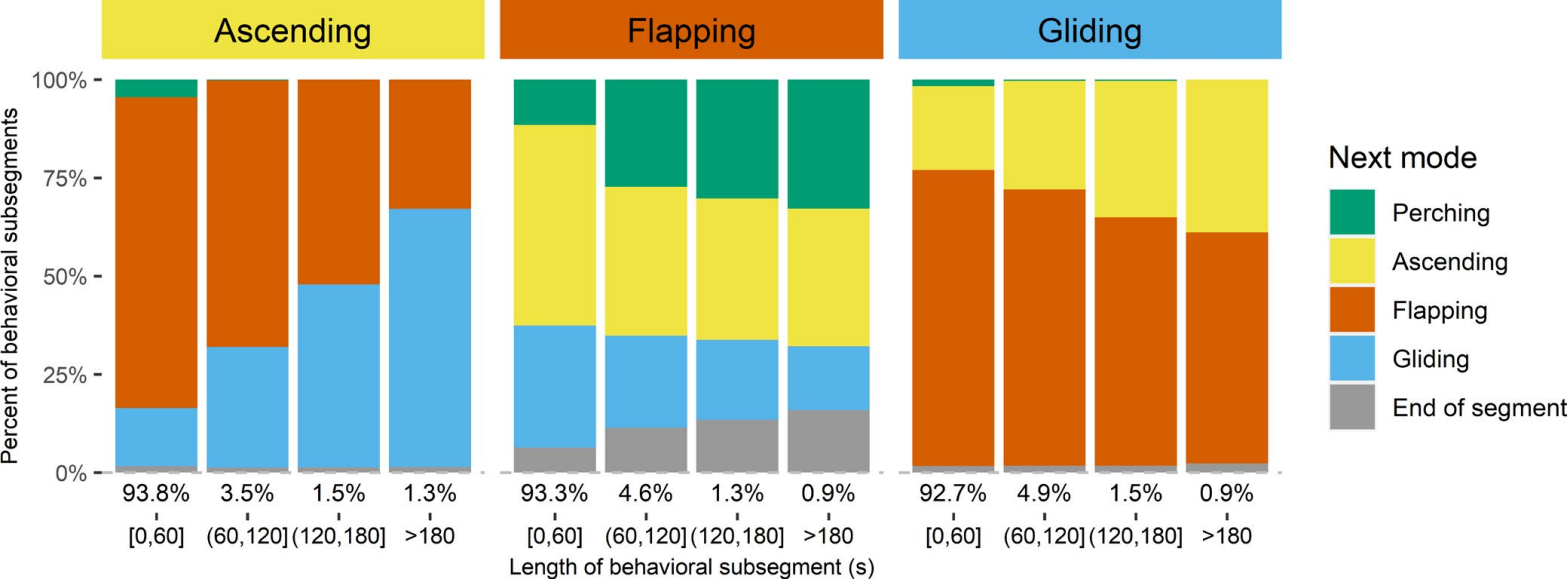
Relationship of length of behavioral subsegments identified in GPS telemetry data and the behavioral mode of the next subsegment. Relationships are shown by behavioral mode. Behavioral classifications were assigned by experts with a strong background in eagle ecology and behavior and associated with *K*‐means clusters of GPS telemetry data from bald eagles in Iowa, USA. See main text for additional details on clustering. Numbers under bars indicate marginal percent of points in each time bracket for the given behavioral mode

Transitions from one type of behavior to another (i.e., change points) can be interpreted to gain biological insight (Table 6). Perching was most likely to occur at the end of a track. Gliding flight was most frequently followed by flapping flight. Flapping and ascending flights most often transitioned between each other, but this relationship depended on the duration of the subsegment (Figure 9). Short periods of ascending flight were most often followed by flapping flight. In contrast, longer ascending flights were most frequently followed by gliding flight.

**TABLE 6:** Transition probabilities for behavior modes of bald eagles

		Subsequent behavior				
		Perching	Ascending	Flapping	Gliding	Segment end
Initial behavior	Perching	–	0.07	0.27	0.01	**0.64**
	Ascending	0.04	–	**0.78**	0.17	0.02
	Flapping	0.13	**0.50**	–	0.30	0.07
	Gliding	0.02	0.22	**0.75**	–	0.02

Note

Behavior labels were assigned by experts with a strong background in eagle ecology and behavior and associated with clusters identified by *K*‐means clustering. Bolded numbers indicate the most likely transition of each initial behavior (i.e., perching is most commonly followed by the end of a subsegment).

## 4 DISCUSSION

### 4.1 Analytic approach and insights into animal movement

Animal movement data are increasingly collected in greater quantity and at shorter time intervals. As these data have the potential to provide important insight for ecologists, selecting the most appropriate analytical method is a crucial component of data interpretation. The choice of an analytical approach should depend on the research objective, with consideration of the complexity, utility, and computational intensity of the method, as well as how those considerations are addressed by packages or software that exist for the implementation (Gurarie et al., 2016).

We have demonstrated use of a specific clustering algorithm, the *K*‐means algorithm, as an efficient method capable of producing biological insight when applied on a GPS‐point basis to vast amounts of short‐interval biologging data consisting of high‐dimensional movement attributes. The ability to cluster points rather than segments is a crucial aspect of our approach. When short segments are abundant and consist of irregular space/time intervals, it may be difficult for segment‐based change‐point approaches such as BCPA, HMM, or BPM to define meaningful change points across the entire data set. For example, the *minimum* segment lengths in the applications considered by Zhang et al. (2015), Langrock et al. (2012), and Gurarie et al. (2009) were, respectively, 266 GPS points from little penguins *Eudyptula minor*, 1427 from American bison, and 30 from northern fur seals *Callorhinus ursinus*. By contrast, in our data set, 88% of our segments were <30 GPS points, 75% of segments were ≤11 points, and the median number of points in a segment was 5. For data like these that are not well suited for segmentation analyses, point‐based *K*‐means clustering proves an effective tool to identify behaviors distinguishable as biologically relevant movement states.

Our approach was unique in considering multidimensional movements and in its computational feasibly. Despite the fact that the two marine species considered in Zhang et al. (2015) and Gurarie et al. (2009) move in three dimensions, neither of these analyses went beyond simple 2‐dimensional linear movement. In contrast, our analysis defined behaviors based on six movement attributes in 3‐dimensional space. Similarly, while existing software packages for fitting many commonly used movement analyses are not equipped to analyze large sets of high‐dimensional movement data, *K*‐means clustering was efficient at clustering nearly two million GPS fixes with each fix characterized by a six‐dimensional movement attribute and did not rely on regular time interval between fixes.

While using solely a quantifiable approach to choosing *K* is appealing, the commonly used elbow method provided no insight into which *K* to choose, and bootstrapping average silhouettes suggested an overly simplistic *K* = 2 as the optimal choice. We therefore relied not entirely on a metric‐based approach, but instead on an exploratory and context‐driven approach for choosing *K* that utilized the bootstrapped silhouettes, a biplot lineup, and additional exploration of the resulting cluster definitions. We feel that this decision is well‐justified. Gurarie et al. (2016) advocated for an approach to movement analysis that is adaptive, iterative, and contains a high exploratory component, in contrast to a prescriptive approach that relies on a large number of *a priori* assumptions. We believe our approach to choosing *K*, by investigating a series of PCA biplots in conjunction with the bootstrapped silhouette averages led us to important biological insight. Specifically, our choice of *K* yielded not just flight vs perching modes, but also described biologically relevant and distinct in‐flight behaviors, while also following Gurarie et al.'s (2016) vision of a less prescriptive, more iterative, and exploratory approach.

Even though our application of *K*‐means clustering was GPS‐point based (rather segment‐based, as in Zhang et al., 2015), we could still gain insight from identifying and exploring characteristics of and transitions between subsegments. Duration of behavioral subsegments (consecutive points assigned to the same cluster) tended to be short and for all moving behaviors that we identified the most frequent subsegment length was 0 s (one GPS point). This is partly because our initial filtering resulted in a large number of short segments, but it is also to be expected from an approach that groups points into clusters based on topology rather than sequentiality. Our results are consistent with field observations of eagles that suggest that flight paths often are comprised of many short, topologically distinct subsegments. For example, in the field, it is common to see an eagle flap a few times to gain altitude, then glide for 10 s, and then repeat this cycle (TAM, TEK, unpublished observations). The advantage of our approach in allowing very short or single‐point behavioral subsegments offers flexibility in accurately characterizing acute behavior changes that are biologically realistic. For example, a momentary leveling off from a gliding descent may be most accurately characterized by very brief flapping flight (Figure 6a; see plot for meters above ground level, where long segments of blue dots are interrupted by one or two orange dots). Similarly, a long flapping flight segment may be interrupted by a very brief ascending segment (Figure 6c; see *X* vs *Y* plot, where long segments of orange dots are interrupted by one or two yellow dots). These acute behavioral changes are identifiable by virtue of the short‐interval data collection and point‐based behavioral classification; consider that BCPA requires segments of at least 30 observations to identify multiple change points (Gurarie et al., 2009).

Simultaneously considering subsegment duration and transition provides additional biological insight beyond that simply from the classification. For example, we noted that ascending flight was most often followed by flapping flight. This initially seemed counterintuitive, as we expected ascending in a thermal to be followed by glides from the thermal. However, this result did not account for the length of the ascending subsegments. When we considered segment length, our results were more biologically sensible (Figure 9). In fact, most of our subsegments were short (95% were <60 s), and it is those short ascending subsegments that were most often followed by flapping flight. However, the longer a bird ascends, the higher it goes and the more likely the ascending flight was followed by gliding flight. This pattern in telemetry data is consistent with our expectation, it makes good intuitive sense, and it matches well with the maps of behavior as well as field observations of birds (TAM, TEK unpublished observations).

A limitation of the *K*‐means algorithm is that every point will be assigned to the single cluster with the multidimensional center to which it is closest. This can result in a great deal of variability of the attributes even among points assigned to the same cluster (which can be seen to some extent in Figure 5), as well as “borderline” points that have been assigned to different clusters but may be more similar to each other than they are to other members of their own cluster. Analysts who are concerned about the “behavioral purity” of points assigned to any one cluster could opt to only include points within a minimum distance from each cluster's centroid, though there is no published recommendation that we could find as to what this minimum distance should be. One possibility for “softer” cluster assignment is to use a method like Gaussian mixtures clustering (Fraley & Raftery, 2002), which assigns to points “cluster responsibilities” that vary between 0 and 1, with higher responsibilities indicating less uncertain cluster membership. However, this method is derived assuming independent observations, which does not hold in the case of biologging data.

### 4.2 Next steps and conclusions

Typical behavioral modeling of telemetry data focuses on identifying segments of consistent behavior and then interpreting those segments. Most approaches are exhibited with two‐dimensional spatial data and may not be useful with data such as those collected from a flying animal. The *K*‐means approach we used is a well‐known statistical tool, but generally, it is not used in a point‐based context to interpret animal tracking data. As we illustrate here, this approach provides a computationally efficient mechanism to rapidly characterize millions of short‐interval animal tracking locations using high‐dimensional movement attributes into biologically relevant behaviors that, if appropriate, can then be used in subsequent behavioral subsegment‐based analyses exploring patterns in sequentiality between and duration of behavioral modes. Whereas existing segmentation‐based approaches are suitable for describing long‐duration behaviors, the availability of short‐interval telemetry data offers the potential for understanding fine‐scale variation in behavior. Point‐based clustering approaches such as *K*‐means provide a way for ecologists to more fully explore their rich data in order to understand intrinsic or extrinsic drivers of those fine‐scale variations.

Describing animal behavior, as we have done here, is only a first step. The next steps would relate these behavioral classifications to the ecological, demographic, or habitat‐related correlates or drivers of that behavior (Nathan, 2008). Our approach lays the groundwork for efficient, effective behavioral classification, setting up subsequent exploration of these important biological questions.

## CONFLICT OF INTEREST

There are no conflicts of interest.

## AUTHOR CONTRIBUTIONS

**Silas Bergen:** Conceptualization (lead); formal analysis (lead); funding acquisition (supporting); writing‐original draft (equal); writing‐review & editing (lead). **Manuela M. Huso:** Formal analysis (supporting); supervision (equal); writing‐original draft (supporting); writing‐review & editing (supporting). **Adam E. Duerr:** Project administration (supporting); supervision (supporting); writing‐original draft (supporting). **Melissa A. Braham:** Data curation (lead); funding acquisition (supporting); writing‐original draft (supporting); writing‐review & editing (supporting). **Todd E. Katzner:** Data curation (supporting); funding acquisition (supporting); project administration (lead); supervision (equal); writing‐original draft (equal); writing‐review & editing (supporting). **Sara Schmuecker:** Funding acquisition (equal); writing‐original draft (supporting). **Tricia A. Miller:** Data curation (supporting); funding acquisition (equal); supervision (equal); writing‐original draft (supporting); writing‐review & editing (supporting).

## Supporting information

Appendix S1

## Data Availability

Braham, M.A., Miller, T.A., Schmuecker, S.J., Duerr, A.E., Bergen, S., and Katzner, T.E., 2021, Data derived from GPS tracking of free‐flying bald eagles (*Haliaeetus leucocephalus*), Iowa, USA: U.S. Geological Survey Data Release, https://doi.org/10.5066/P9HZZZ26. An example data file and R code for carrying out the *K*‐means clustering and subsequent analyses described here are available at https://github.com/silasbergen/kmeans_behavioral_classification.
