# Supplementary material for: Classifying behavior from short‐interval biologging data: An example with GPS tracking of birds
Source: Ecol Evol. 2022 Feb 7;12(2):e08395. doi: 10.1002/ece3.8395 (PMC8819645; doi:10.1002/ece3.8395)
Supplement: Supplementary file 1 — Appendix S1 [file ECE3-12-e08395-s001.docx]

***Supplemental information for Bergen et al.***

**Classifying behavior from short-interval bio-logging data: an example with GPS tracking of birds**

**Some background on the eagles used in this study**

At the time of tagging, birds ranged in age from nestling (n = 42), to juveniles (3), to subadults (2), to adult (10; SI Table 1). Of these, 4 died before fledging and 39 did not spend time in Iowa, leaving a final sample of 57 individuals from which telemetry data were gathered within the state. As the number of tagged birds increased annually, we collected more data points in each year of the study (Table 2).

**Information on selection of variables used in the study**

KPH and Sn are both measures of velocity; their correlation coefficient is 0.86. We included both of them because differences between the two can be important for identifying certain behaviors. KPH measures the speed at which the bird was traveling when the GPS point was collected and Sn is the average horizontal speed between two points. In a situation where a bird was exiting a directional flight and transitioning into an ascending flight within a thermal, the KPH (GPS speed) would be higher than the speed between points (Sn) (Katzner et al., 2015).

We included absolute value of turning angle instead of the raw angle because the size of the angle is more important to our analysis than is identifying whether the bird was turning in a clockwise or counterclockwise direction.

Vertical rate measures how quickly the bird’s altitude is increasing or decreasing. We included the absolute value of this variable to characterize whether the bird was in level or non-level flight.

**Literature Cited**

Katzner, T. E., Turk, P. J., Duerr, A. E., Miller, T. A., Lanzone, M. J., Cooper, J. L., Brandes, D., Tremblay, J. A., & Lemaître, J. (2015). Use of multiple modes of flight subsidy by a soaring terrestrial bird, the golden eagle *Aquila chrysaetos*, when on migration. *Journal of the Royal Society Interface*, *12,* 20150530. https://doi.org/10.1098/rsif.2015.0530

**SI Table 1.** Number of bald eagles tagged and number of individuals tracked with GPS telemetry, organized by bird age. Many birds were tagged as nestlings but since nestlings don’t fly, none of their tracking data were relevant to the analysis presented here. Most birds were captured in one stage and tracked into one or two other life stages, hence the counts for each age class often are larger than the numbers of individuals tagged in each age class.

| Bird Age | Number captured | Individuals tracked |
| --- | --- | --- |
| Nestling | 42 | 28 |
| Juvenile | 3 | 39 |
| Subadult | 2 | 23 |
| Adult | 10 | 11 |
| **Total** | **57** | **101** |


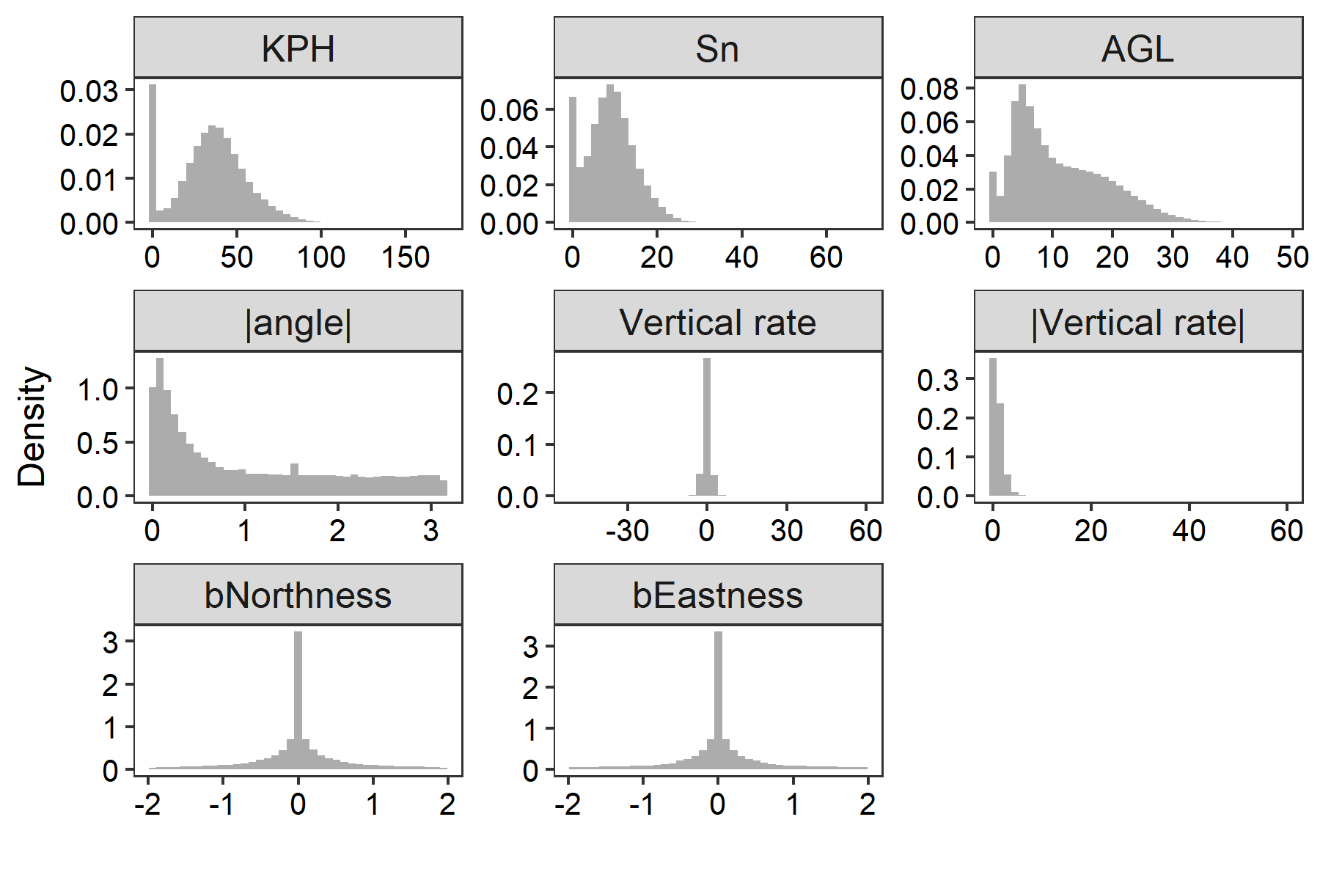


**SI Figure 1*:*** Distributions of eight focal variables associated with GPS data collected from bald eagles in Iowa, USA. Focal variables were standardized (after square-root transform when necessary to reduce skewness) then used as input into a *K*-means cluster analysis to classify flight behavior of these birds. See text for details on analysis.


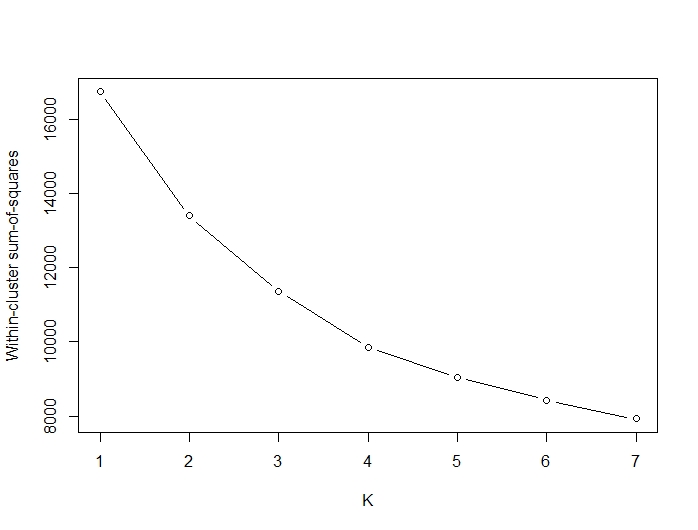


**SI Figure 2:** Plot of within-cluster sum-of-squared distances between each point and the cluster centroid as a function of number of specified clusters *K.* Cluster centroids were determined by a *K*-means analysis of standardized GPS telemetry data collected from bald eagles in Iowa, USA. See main text for additional details on data collection and analysis.


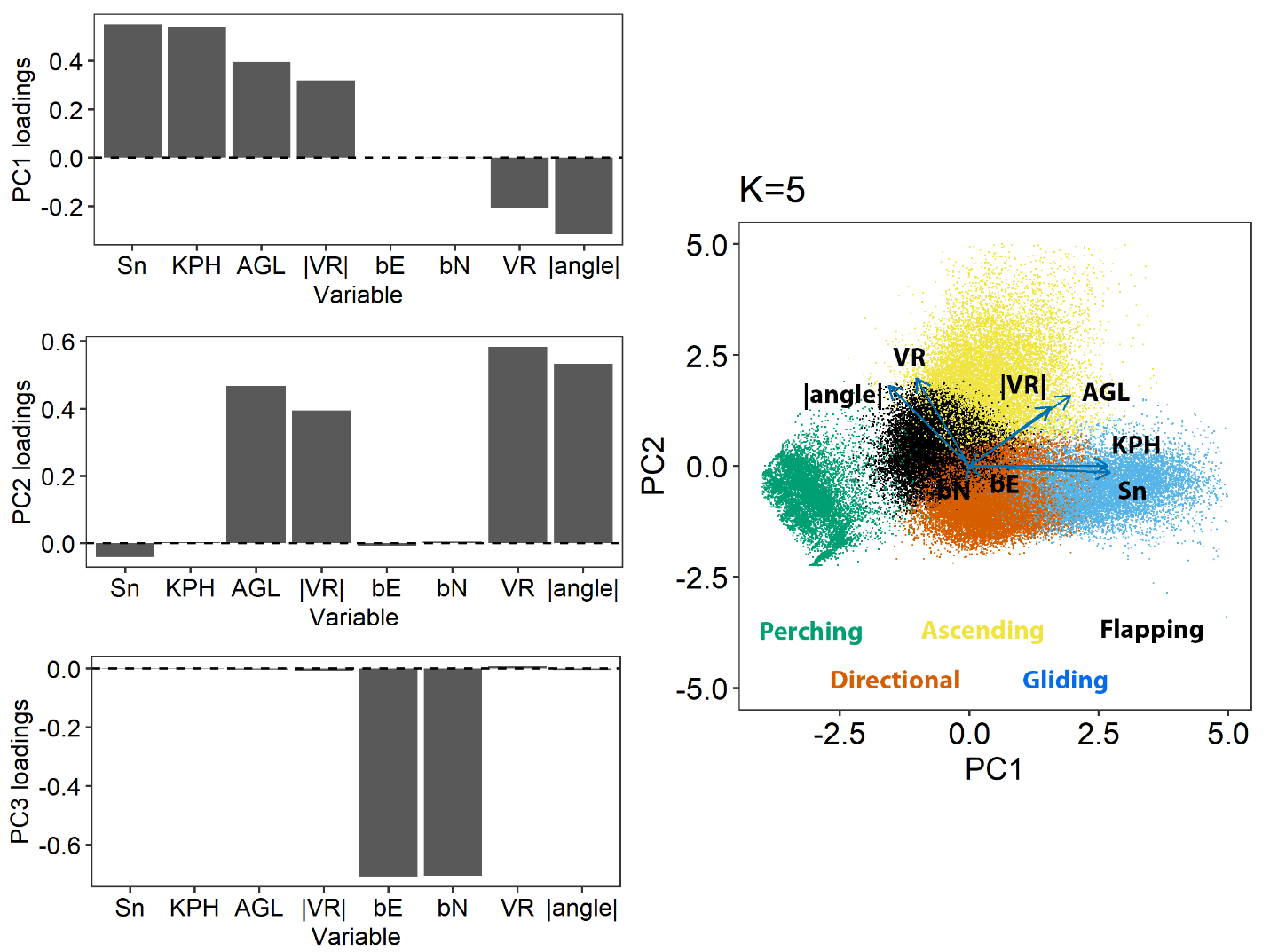


**SI Figure 3:** Loadings and biplot from principal component analysis of GPS telemetry data collected from bald eagles in Iowa, USA. Input data were the 8 focal variables shown in Table 1 in the main text. The first three components accounted for, respectively, 34%; 16%; and 12% of the total variability in the covariates. Points on biplot are from a representative bird and are color-coded by cluster membership assignment for *K*=5 clusters.


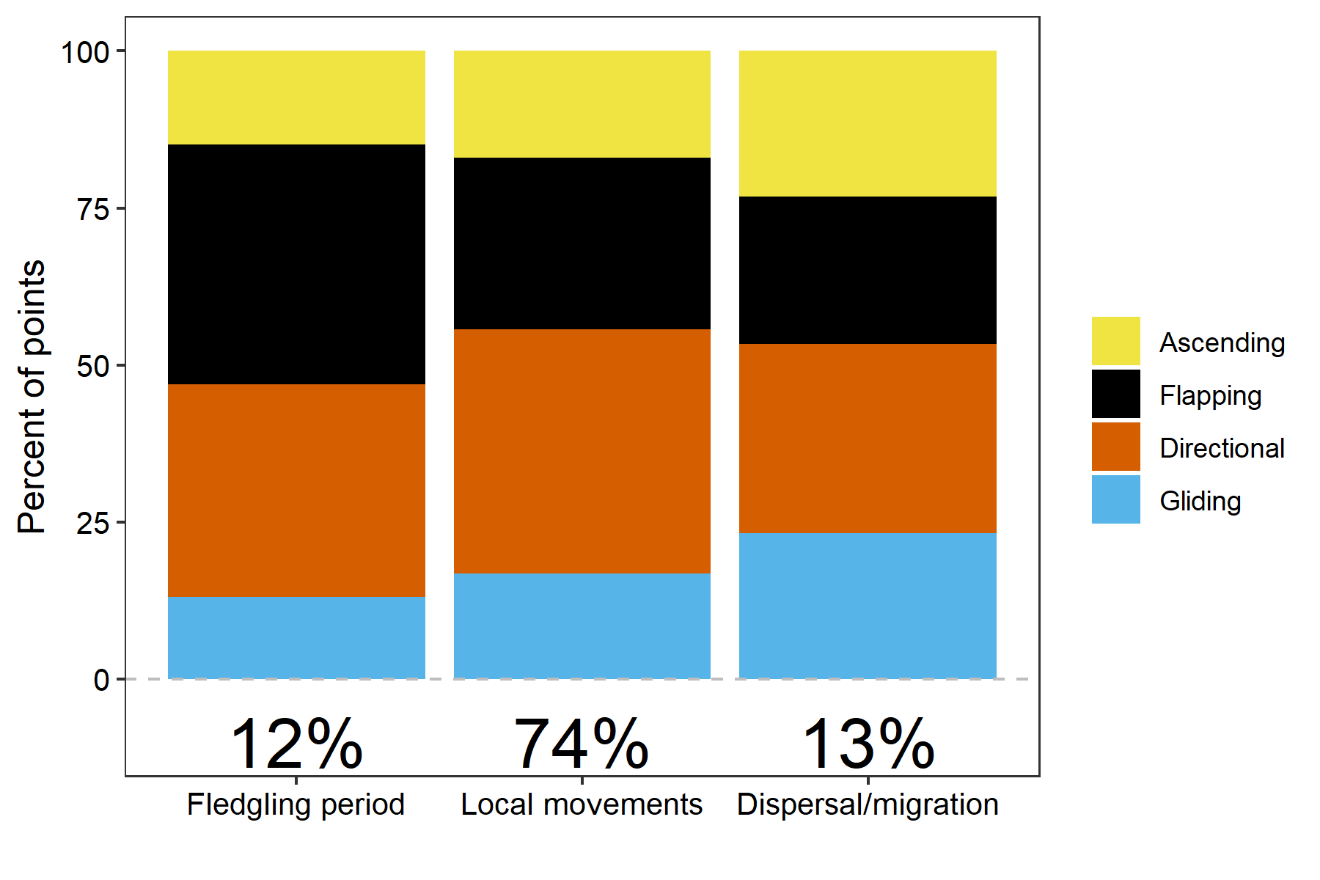


**SI Figure 4:** Relationship of behavioral classifications with life stages of eagles. Behavioral classifications were assigned by experts with a strong background in eagle ecology and behavior and associated with *K*-means clusters of GPS telemetry data from bald eagles in Iowa, USA. Life stages of eagles were determined by gross movement characteristics of birds (i.e., were their movements migratory or local in nature). See main text for additional details on clustering and assignment to life stages. Bold numbers under bars indicate marginal percent of points in each flight stage.


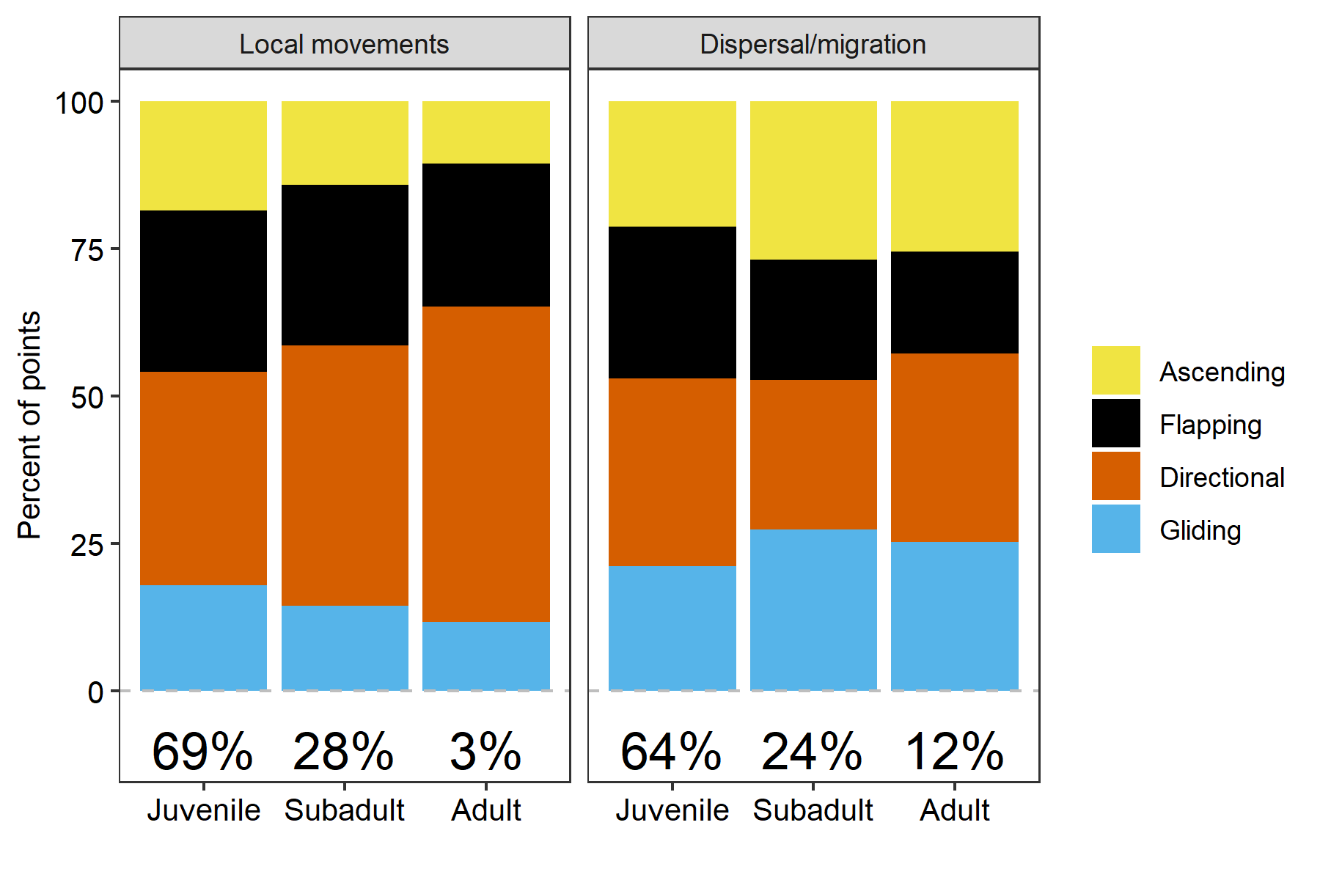


**SI Figure 5:** Relationship of behavioral classifications with age classes and life stages of eagles. Behavioral classifications were assigned by experts with a strong background in eagle ecology and behavior and associated with *K*-means clusters of GPS telemetry data from bald eagles in Iowa, USA. Life stages of eagles were determined by gross movement characteristics of birds (i.e., were their movements migratory or local in nature). Ages were estimated when eagles were marked. See main text for additional details on clustering and assignment to age classes and life stages. Bold numbers under bars indicate marginal percent of points in each age class and of each flight stage.


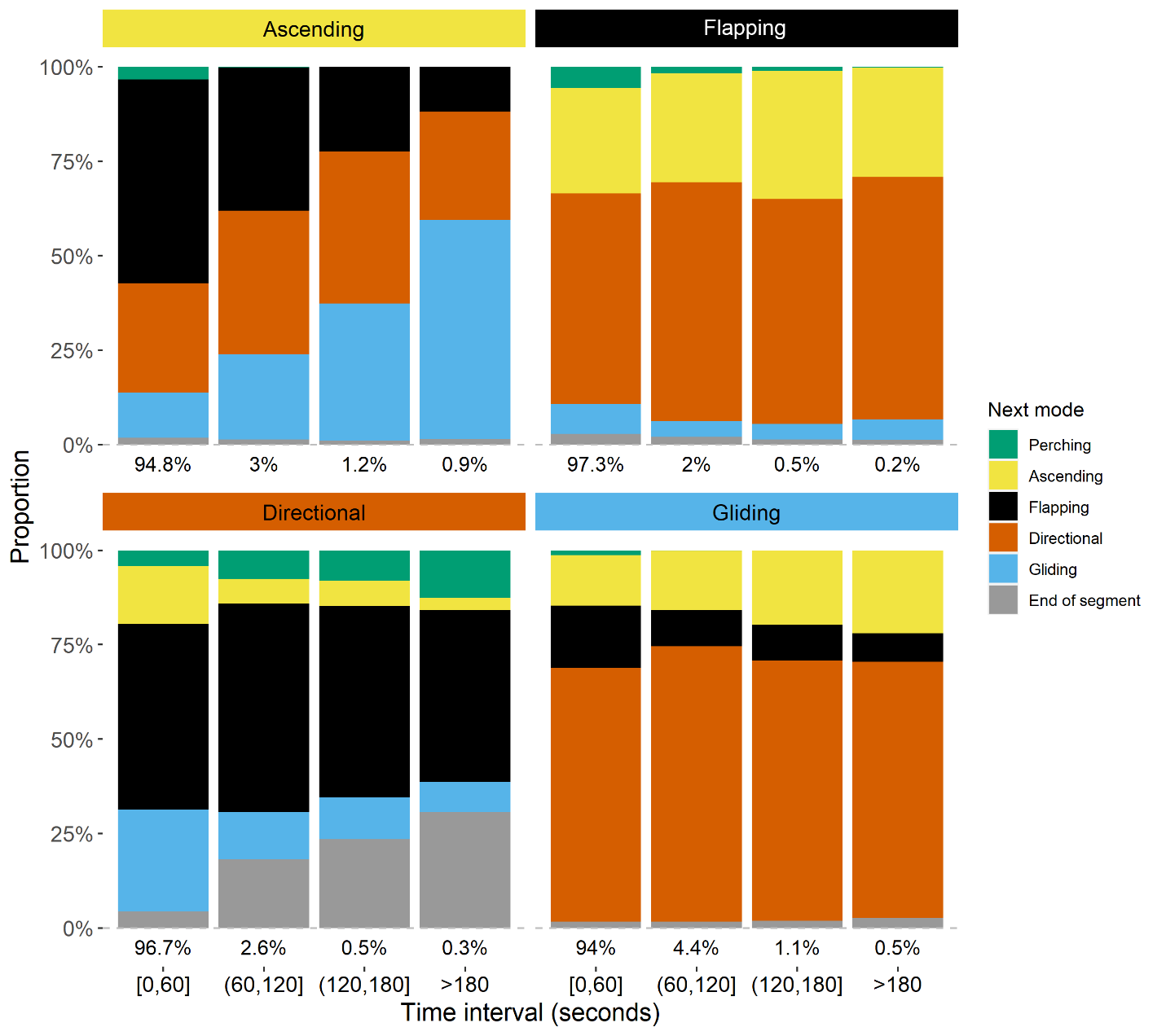


**SI Figure 6:** Relationship of length of behavioral subsegments identified in GPS telemetry data and the behavioral mode of the next subsegment. Relationships are shown by behavioral mode. Behavioral classifications were assigned by experts with a strong background in eagle ecology and behavior and associated with *K*-means clusters of GPS telemetry data from bald eagles in Iowa, USA. See main text for additional details on clustering. Numbers under bars indicate marginal percent of points in each time bracket for the given behavioral mode.
